# Bacteria-Inspired Synthesis of Silver-Doped Zinc Oxide Nanocomposites: A Novel Synergistic Approach in Controlling Biofilm and Quorum-Sensing-Regulated Virulence Factors in *Pseudomonas aeruginosa*

**DOI:** 10.3390/antibiotics14010059

**Published:** 2025-01-09

**Authors:** Abirami Karthikeyan, Manoj Kumar Thirugnanasambantham, Fazlurrahman Khan, Arun Kumar Mani

**Affiliations:** 1 Department of Chemistry and Biosciences, Srinivasa Ramanujan Centre, SASTRA Deemed University, Kumbakonam 612001, India; abirami.karthi1@gmail.com (A.K.); manojkumar@scbt.sastra.ac.in (M.K.T.); 2 Ocean and Fisheries Development International Cooperation Institute, Pukyong National University, Busan 48513, Republic of Korea; fkhan055@pknu.ac.kr; 3 International Graduate Program of Fisheries Science, Pukyong National University, Busan 48513, Republic of Korea; 4 Marine Integrated Biomedical Technology Center, The National Key Research Institutes in Universities, Pukyong National University, Busan 48513, Republic of Korea; 5 Research Center for Marine Integrated Bionics Technology, Pukyong National University, Busan 48513, Republic of Korea

**Keywords:** Ag-ZnO NCs, anti-quorum sensing, *Pseudomonas aeruginosa*, anti-biofilm activity

## Abstract

Multidrug-resistant *Pseudomonas aeruginosa* infections pose a critical challenge to healthcare systems, particularly in nosocomial settings. This drug-resistant bacterium forms biofilms and produces an array of virulent factors regulated by quorum sensing. In this study, metal-tolerant bacteria were isolated from a metal-contaminated site and screened for their ability to synthesize multifunctional nanocomposites (NCs). Rapid color changes in the reaction solution evidenced the biotransformation process. The potent isolated *Bacillus cereus* SASAK, identified via 16S rRNA sequencing and deposited in GenBank under accession number MH885570, facilitated the microbial-mediated synthesis of ZnO nanoparticles and silver-doped ZnO NCs. These biogenic nanocomposites were characterized using UV-VIS-NIR spectroscopy, FTIR, XRD, zeta potential, HRTEM, FESEM, and EDX analyses. At a sub-MIC concentration of 100 µg/mL, 2% Ag-ZnO NCs effectively inhibited virulent factor production and biofilm formation in *P. aeruginosa* without affecting bacterial growth. Notably, there was a significant reduction in violacein pigment (96.25%), swarming motility, and pyocyanin concentration (1.87 µg/mL). Additionally, biofilm formation (81.1%) and EPS production (83.9%) using *P. aeruginosa* were substantially hindered, along with reduced extracellular protease activity, as indicated by zone formation (from 2.3 to 1.8 cm). This study underscores the potential of Ag-ZnO NCs as promising agents for combating quorum sensing-mediated virulence in chronic infections caused by multidrug-resistant *P*. *aeruginosa.*

## 1. Introduction

Nanobiotechnology stands at the forefront of rapid scientific advancement today, finding diverse applications across various fields. Combining biology with nanotechnology, nanobiotechnology harnesses nanoscale materials, typically ranging from 1 to 100 nm in size, to combat human diseases and disorders through targeted biological applications [1]. Nanocomposites are an innovative class of unique materials manufactured by combining one or more distinct components at the nanoscale with the intent to regulate and build new and enhanced shapes and features. Bioengineered nanocomposites are essential because of their intrinsic qualities, such as high surface-area-to-volume ratio and multifunctionality, as well as designed properties, such as porosity, stability, and permeability [2]. Among the various approaches used for synthesizing nanocomposites, such as physical, chemical, and biological methods, the fabrication of nanocomposites using microorganisms stands out for its cost-effectiveness, high yield, minimal energy consumption, and non-toxicity [3]. The bacteria-inspired synthesis of silver nanoparticles has recently garnered significant attention due to their potent antimicrobial properties and extensive applications in medical industries [4]. Natural sources used in biological methods ensure safety, affordability, sustainability, and eco-friendliness [5]. Microbes involved in nanoparticle synthesis produce extracellular reductase enzymes that convert metal ions into nanoparticles quickly and uniformly. This microbial-mediated synthesis yields highly biocompatible nanoparticles and exhibits low polydispersity, benefiting from various enzymes and proteins that act as stabilizing agents [6]. Doping silver ions with other metal oxide nanoparticles synergistically enhances catalytic activity through ion transformation methods, such as incorporating silver ions into zinc oxide (ZnO) nanoparticles. ZnO nanoparticles are renowned for their non-toxicity and environmental compatibility, making them particularly suited for doping processes with silver ions, which are highly favored for their antimicrobial efficacy [7,8]. Bacterial-mediated biotransformation processes are utilized to reduce silver nitrate to silver nanoparticles, leveraging bacteria, yeast, fungi, and plants for their bio-reducing capabilities [9]. The rise of drug-resistant bacteria underscores the urgent need for novel antimicrobial agents or materials to combat pathogenic microorganisms. Current antibiotics face increasing resistance, with up to 50% of hospitalized patients affected by multidrug-resistant bacteria, posing a severe threat to medical industries [10,11]. *Pseudomonas aeruginosa*, a prominent pathogen in nosocomial infections among immunocompromised patients, particularly those with cystic fibrosis, is known for its robust biofilm formation facilitated by quorum sensing mechanisms [12,13]. Quorum sensing (QS) enables bacteria to coordinate and regulate biofilm production through signaling molecules like peptides and acyl homoserine lactones, protecting them from environmental stress and antibiotics [14]. Researchers actively explore nanostructures to inhibit QS molecules, effectively preventing biofilm formation [15]. ZnO and silver nanoparticles have demonstrated strong anti-quorum sensing activity by down-regulating quorum sensing-regulated genes, inhibiting virulence, antimicrobial resistance, and biofilm formation [16,17,18]. In this study, pure and silver-doped ZnO nanocomposites were synthesized using bacteria isolated from metal-polluted environments, utilizing bacterial enzymes and metabolites as reducing and capping agents. This investigation represents the first exploration of the anti-quorum sensing efficacy of silver-doped ZnO NCs against multidrug-resistant *Pseudomonas aeruginosa*.

## 2. Results

### 2.1. Isolation, Screening, and Identification of Metal-Tolerant Strains

**Figure 1:**
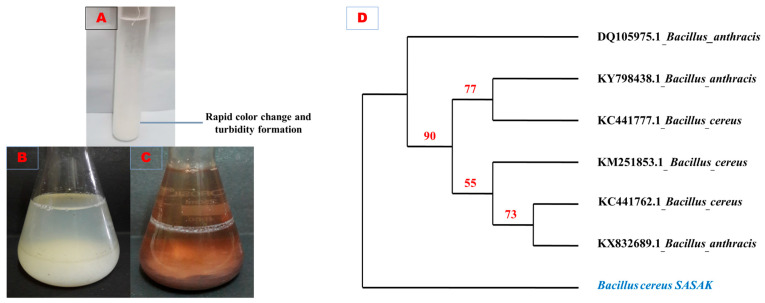
Screening and molecular identification of potent bacterial strain for the synthesis of Ag-ZnO NCs. (**A**) Screening of isolated bacteria (SASAK) for the synthesis of Ag-ZnO NCs and bacterial-inspired synthesis of Ag-ZnO NCs, (**B**) initial color change, (**C**) final color change, and (**D**) phylogenetic tree of isolated bacteria (*Bacillus cereus* SASAK).

A metal-tolerant bacterium was isolated from soil samples using enrichment techniques and designated as SASAK. The quick color shift and turbidity growth in the screening medium indicate the great ability of the isolated bacteria to synthesize NCs (Figure 1A). The Ag-ZnO NCs were synthesized using the isolated *Bacillus cereus* SASAK.

The isolated bacteria were cultivated in a sterile solution containing zinc acetate dihydrate and silver nitrate as a substrate for the synthesis of NCs. During incubation, instant color change occurred in the flask (1–2 h), transitioning from white to brown, indicating the biotransformation process, as depicted in Figure 1B,C. Morphologically, the isolated bacterial strain exhibited flat, smooth, and large round colonies and was characterized as a Gram-positive, rod-shaped, motile, and spore-producing organism. Further, this potent heavy metal-tolerant bacterium was characterized by molecular detection through 16S rRNA sequencing to assess its similarity to related species. The isolated SASAK bacteria yielded a nucleotide sequence of approximately 1258 bp, confirming its identity. The 16s rRNA sequencing revealed that the isolated SASAK strain shows the highest homology similarity (99%) for *Bacillus cereus*. CLC MAIN Workbench 4 software was used to create the phylogenetic tree in order to comprehend the evolutionary relationships between the strains that synthesize efficient nanocomposites and other related organisms (Figure 1D). The nucleotide sequence data of the isolated bacteria were submitted to the NCBI Database and GenBank, receiving the unique identification number MH885570.

### 2.2. Characterization of Pure and Ag-ZnO NCs

#### 2.2.1. UV–Visible NIR Spectrum

**Figure 2:**
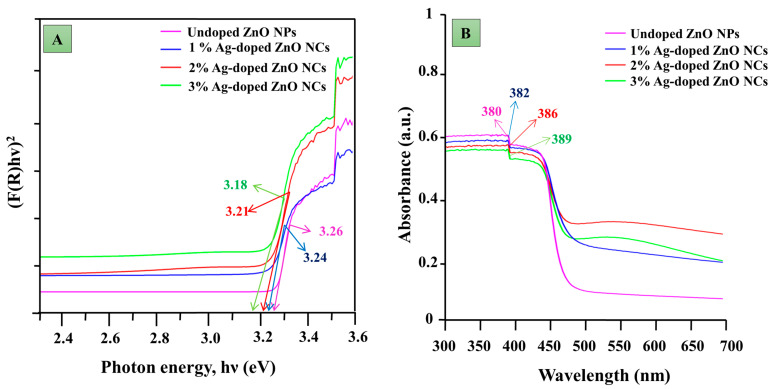
UV-vis NIR spectrum of undoped ZnO NPs and Ag-ZnO NCs. (**A**) Bandgap spectra and (**B**) UV-vis absorption spectra.

UV–visible NIR spectroscopy was employed to measure the absorbance and bandgap energy of the nanocomposites. Figure 2B illustrates that the absorbance value for ZnO nanoparticles was 380 nm, while for Ag-ZnO NCs, the absorbance values were 382 nm, 386 nm, and 389 nm for 1%, 2%, and 3% silver concentrations, respectively. The bandgap energy was determined from these wavelength values and calculated using the Kubelka–Munk equation (Equation (1)).

(1) $$F\left(R\right)=\frac{(1-{R)}^{2}}{2R}$$

where ‘R’ is the reflectance value. The bandgap energy was examined using photon energy with the variation in the Kubelka–Munk equation. Wavelength values are correlative to the intersection points of a horizontal and vertical part of spectra using the following equations (Equation (2) and (3)).

(2) Eg=hc/λ

(3) $$Eg=1240/\lambda_{max}eV$$

where ‘E_g_’ denotes the bandgap energy (eV), h is the plank constant (6.626 × 10^−34^ Js), c denotes the velocity of the light (3 × 10^8^ m/s), λ is the wavelength, and λ_max_ is the maximum wavelength at the intersection point.

The data represented in Figure 2A show the pure bandgap energy (3.26 eV), and Ag-doped ZnO NCs (1, 2, and 3%) were noted to be 3.24 eV, 3.21 eV, and 3.18 eV, respectively. The bandgap energy declined in Ag-ZnO NCs in a dose-dependent manner in contrast to pure ZnO NPs.

#### 2.2.2. Measuring the Size and Stability of NCs

**Figure 3:**
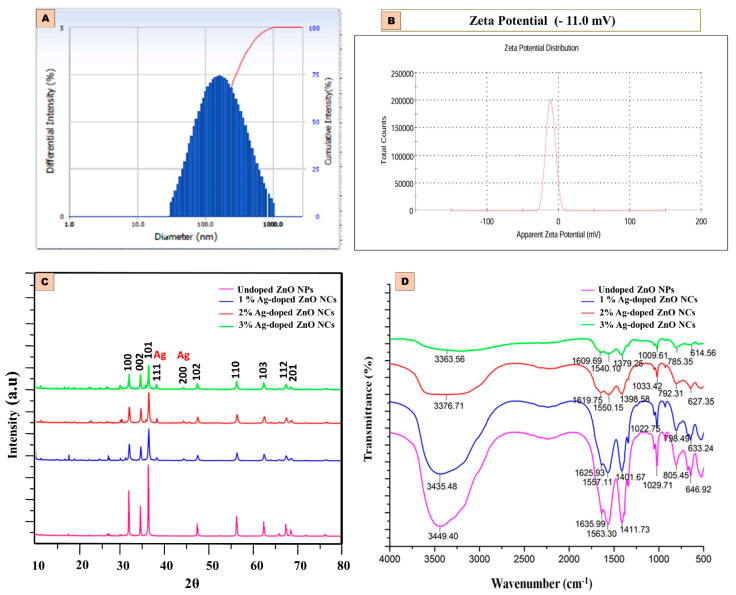
Characterization of NCs (**A**). Histogram showing the particle size analysis of 2% Ag-ZnO NCs, (**B**) the zeta potential distributions of 2% Ag-ZnO NCs, (**C**) the XRD spectra of undoped ZnO NPs and Ag-ZnO NCs, and (**D**) the FTIR spectrum of undoped ZnO NPs and Ag-ZnO NCs.

The average particle size of 2% Ag-doped ZnO NCs produced by isolated *Bacillus cereus* SASAK was 137.1 nm (Figure 3A). The hydrodynamic size of these nanocomposites slightly increased due to the accumulation of biometabolites produced by the bacteria during synthesis.

The findings reveal that the generated NCs are equally monodispersed, with a polydispersity index of 2% Ag-doped ZnO NCs being approximately 0.26. Furthermore, the zeta potential analysis was employed to assess the potential stability and surface charge of the Ag-doped ZnO NCs. As shown in Figure 3B, the zeta potential value of 2% Ag-doped ZnO NCs was measured at −11.0 mV.

#### 2.2.3. XRD Analysis of Ag-ZnO NCs

The X-ray diffraction (XRD) patterns of bacterial-mediated synthesized ZnO NPs and Ag-ZnO NCs are depicted in Figure 3C. The characteristic XRD pattern confirms that the products exhibited a hexagonal wurtzite phase. Broad and sharp absorption peaks at 2θ values of 31.8°, 34.5°, and 36.3° corresponded to the (100), (002), and (101) planes of ZnO, respectively. Additional weaker peaks observed at 2θ values of 47.6°, 56.6°, 62.9°, 67.98°, and 69.1° corresponded to the (102), (110), (103), (112), and (201) planes. Two secondary absorption peaks at 2θ = 38.2° and 44.3°, corresponding to the (111) and (200) planes, respectively, indicate the presence of silver nanoparticles. The shift in these peaks suggests the effective doping of ZnO with varying concentrations of silver compared to undoped ZnO peaks.

#### 2.2.4. FTIR Analysis of Ag-ZnO NCs

Figure 3D shows that the bacterial-inspired synthesis of pure and Ag-doped ZnO NCs possessed broad absorption peak bands in FTIR at 3449.40 cm^−1^ and 3376.71 cm^−1^ corresponding to O-H stretching vibration; peak bands at 1635.99 cm^−1^, 1619.75 cm^−1^, and 1563.30 cm^−1^ corresponded to the C=C stretching vibration; peak bands at 1029.71 cm^−1^ and 1033.42 cm^−1^ corresponded to the C-N stretching vibration; and peak bands at 805.45 cm^−1^ and 792.31 corresponded to the C-H bending mode. In 2% Ag-ZnO NCs, the peaks located at 1550.15 cm^−1^ corresponded to N-O stretching and those at 1398.58 cm^−1^ corresponded to the C-H bending mode. The peak located at 1411.73 cm^−1^ can be attributed to the O-H bending mode in pure ZnO NPs. The presence of metal oxide was proved by a peak of 646.92 cm^−1^, confirming the ZnO NPs. Silver nanoparticles were confirmed by the peaks at 614.56 cm^−1^ to 633.24 cm^−1^. A small shift in bands, as well as alterations in the bond length, indicated the doping location.

#### 2.2.5. HRTEM, FESEM, and EDX Analysis of Ag-ZnO NCs

**Figure 4:**
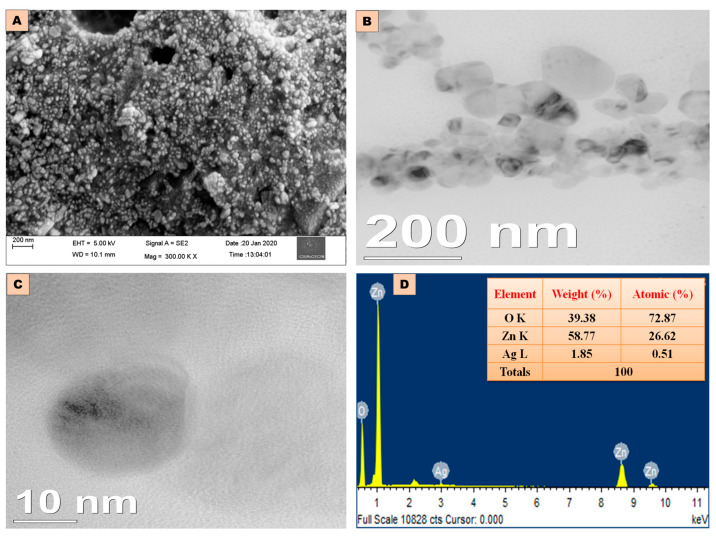
Morphological and elemental composition of 2% Ag-ZnO NCs (**A**). FESEM analysis (**B**) and (**C**) size analysis of NCs using HRTEM, and (**D**) EDX analysis of NCs.

Figure 4A depicts spherical-shaped agglomerated Ag-ZnO NCs at various magnifications. HRTEM analysis shown in Figure 4B,C confirms that the nanocomposites were agglomerated, spherical, and measured 32 ± 2 nm in size. The EDX spectrum in Figure 4D provides elemental and compositional information of 2% Ag-doped ZnO NCs. It also confirms the presence of Zn, Ag, and oxygen (O) elements, with their respective weight (%) and atomic (%) compositions. Peaks in the EDX spectrum were located at 3 keV for Ag, 0.55 keV for O, and 1.02 keV, 8.7 keV, and 9.5 keV for Zn elements.

### 2.3. Antibiotic Susceptibility Test Against Pathogen (P. aeruginosa)

**Figure 5:**
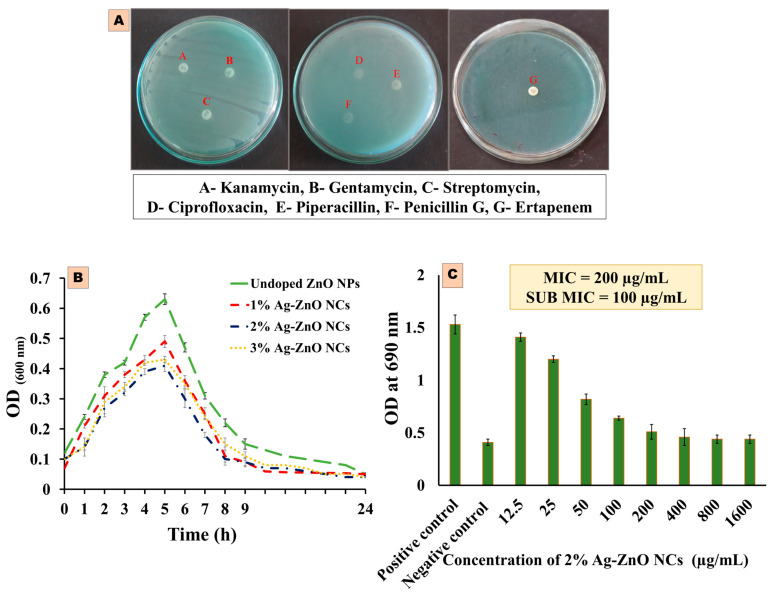
(**A**) Antibiotic susceptibility test against the hospital-acquired test pathogen *P. aeruginosa*; (**B**) the effect of pure and Ag-ZnO NCs on the cell viability of *P. aeruginosa*; and (**C**) the determination of the MIC and sub-MIC of 2% Ag-ZnO NCs, including the positive control (without NCs) and negative control (0.5 µg/mL of colistin).

The antibiotic susceptibility test was conducted using the disc diffusion method to assess the multidrug-resistant profile of a selected pathogen. The tested pathogens showed resistance to all seven antibiotics, including gentamicin, streptomycin, kanamycin, ciprofloxacin, piperacillin, penicillin G, and ertapenem. No inhibition zones were observed around the antibiotic discs (Figure 5A). These results indicate that the hospital-acquired *P. aeruginosa* strain exhibited multidrug resistance against the antibiotics tested.

### 2.4. MIC and Sub-MIC Determination

The initial test aimed to assess the effect of the antimicrobial susceptibility of pure and Ag-ZnO NCs on the survival of the test pathogen using the broth tube dilution method with varying concentrations over time intervals. The results indicated that 2% Ag-ZnO NCs exhibited the highest inhibitory effect on cell viability compared to pure ZnO NPs and other concentrations of Ag-ZnO NCs (Figure 5B). The MIC concentration of 2% Ag-doped ZnO NCs against *P. aeruginosa* was determined to be 200 µg/mL, with a sub-MIC concentration of 100 µg/mL, as shown in Figure 5C. This sub-MIC level was subsequently used as the standard concentration for anti-quorum sensing assays. MIC and sub-MIC values were determined based on optical density measurements at 600 nm. Typically, concentrations of Ag-doped ZnO NCs above the MIC value of 200 µg/mL effectively eradicated the test pathogen by increasing microbial cell permeability, leading to the leakage of cellular contents and damage to biomolecules within the cells.

### 2.5. Antiquorum Sensing Activity of Ag-ZnO NCs

#### 2.5.1. Violacein Production Inhibition Assay

**Figure 6:**
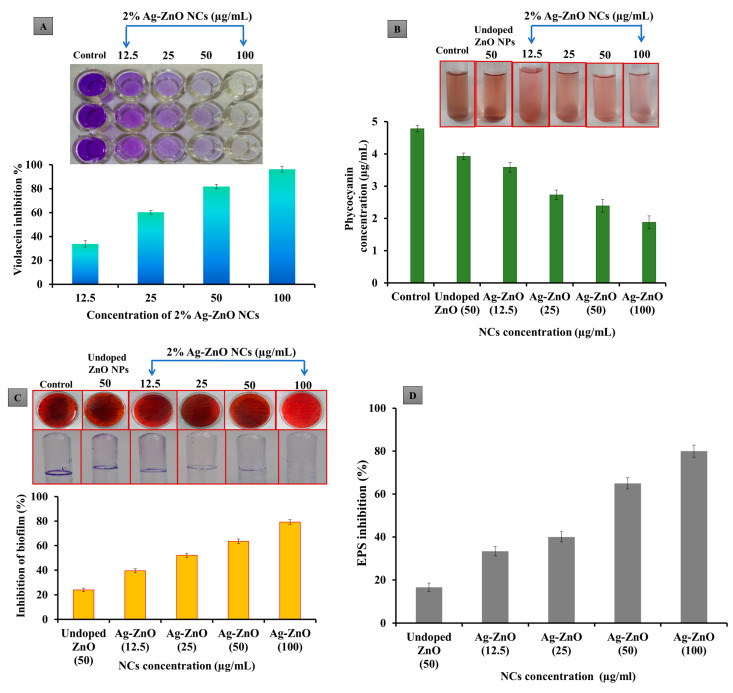
Antiquorum sensing activity of NCs. (**A**) The inhibition of the violacein pigment in bioreporter strain *C. violaceum* using 2% Ag-ZnO NCs, (**B**) the pyocyanin inhibition assay, and (**C**) the inhibition of biofilm formation using undoped ZnO NPs and 2% Ag-ZnO NCs using the Congo red assay and tube assay, and (**D**) the inhibition of EPSs using undoped ZnO NPs and 2% Ag-ZnO NCs.

Sub-MIC levels of Ag-ZnO NCs were employed to evaluate their effect on inhibiting violacein pigment production in *Chromobacterium violaceum*. Figure 6A illustrates the inhibition activity of Ag-ZnO NCs against the bioreporter strain *C. violaceum*, where the suppression of violacein production showed a direct concentration-dependent relationship compared to the control. The maximum reduction of violacein production, 96.25%, was observed at 100 µg/mL concentrations of Ag-ZnO NCs.

#### 2.5.2. Inhibition of Pyocyanin Using Ag-ZnO NCs

The impact of NCs on pyocyanin production was assessed using an overnight culture of *P. aeruginosa* treated with ZnO NPs and 2% sub-MIC of Ag-doped ZnO NCs. As illustrated in Figure 6B, the production of pyocyanin in *P. aeruginosa* decreased in the range of 17–60% when supplemented with ZnO NPs and 2% sub-MIC of Ag-doped ZnO NCs compared to the control. The most significant reduction in pyocyanin production (1.87 µg/mL) against multidrug-resistant (MDR) *P. aeruginosa* was observed at 100 µg/mL of 2% sub-MIC of Ag-doped ZnO NCs.

#### 2.5.3. Ag-ZnO NCs Inhibit Initial-Stage Biofilms

The crystal violet assay evaluated the antibiofilm activity of 2% Ag-ZnO NCs against hospital-acquired multidrug-resistant (MDR) *P. aeruginosa*. Figure 6C illustrates that the maximum reduction in biofilm formation, up to 81.1%, was observed at a 100 µg/mL concentration. The assay confirmed that NCs exhibited minimal impact on the growth of the test pathogens, as evidenced by the viable bacterial growth on agar plates. The Congo red assay was conducted to evaluate the impact of 2% Ag-ZnO NCs on biofilm production using multidrug-resistant (MDR) *P. aeruginosa*. On NC-treated plates, colonies exhibited a pink coloration, contrasting with the black colonies on control plates, which indicated a complete reduction in biofilm production.

#### 2.5.4. EPS Reduction and Quantification Assay Using Ag-ZnO NCs

Figure 6D indicates a notable reduction in EPS production, with the maximum inhibition observed at 100 µg/mL. *P. aeruginosa* EPS production was measured in response to 2% Ag-ZnO NCs, and the highest suppression of EPS production was determined to be 83.9%.

#### 2.5.5. Ag-ZnO NCs Disrupt Swarming Motility

**Figure 7:**
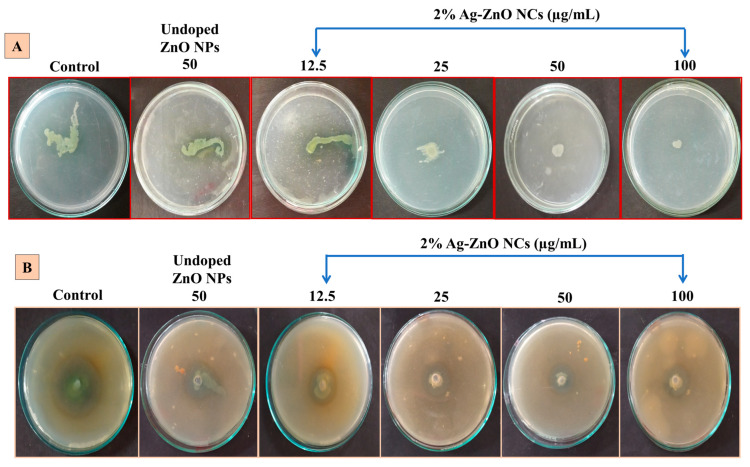
(**A**) The inhibition of swarming motility and (**B**) protease inhibition assay.

The inhibitory effects of ZnO NPs and sub-MIC concentrations of 2%Ag-ZnO NCs at various levels were tested on the swarming movement of *P. aeruginosa.* Figure 7A depicts the complete suppression of swarming activity with 2% Ag-ZnO NCs at a concentration of 50 µg/mL compared to the control.

#### 2.5.6. Ag-ZnO NCs Attenuate the Protease Activity

The protease assay assessed the impact of NCs on extracellular protease production using *P. aeruginosa*. The NCs effectively decreased proteolytic activity by inhibiting protease enzymes. The control plates exhibited a clear hydrolysis zone of 4 cm, whereas the test plates treated with NCs showed reduced zone diameters ranging from 2.3 to 1.8 cm, as shown in Figure 7B. At higher concentrations (100 µg/mL) of nanocomposites, the inhibition zone was narrower (1.8 cm).

## 3. Discussion

*Pseudomonas aeruginosa* develops antibiotic resistance through innate, acquired, or adaptive methods, and conventional antibiotics are ineffective in treating *P. aeruginosa*. It also contributes to the emergence of multidrug-resistant strains [19]. In this study, we used nano-biotechnology to control the biofilm and virulence factor of MDR *P. aeruginosa*. The strain isolated from the metal contaminant site was identified through the PCR amplification of 16S rRNA fragments [20]. Additionally, the isolated metal-tolerant *Bacillus cereus* strain demonstrated the capability to produce hydrolytic enzymes that facilitate the synthesis of metal nanoparticles [21].

A variety of analytical techniques were used to characterize the synthesized nanoparticles. The increase in the absorption of Ag-ZnO nanoparticles showed a concentration-dependent trend attributed to electron transitions between the valence and conduction bands [22]. The maximum absorption of undoped nanoparticles in UV–visible spectroscopy was observed between 320 and 390 nm [23], whereas for Ag-doped ZnO nanoparticles, it spanned from 200 to 800 nm [24]. A reduction in the bandgap energy was purely due to the recombination of the electron shuttle that occurred between the semiconductors of ZnO to the metal ions (Ag) [25] and also due to the creation of oxygen vacancies in the semiconductor which triggered the transition of electrons between the various bands [26]. Additionally, the concentration of silver ions embedded in the ZnO lattice sites influenced the surface morphology and contributed to the size increase in the nanoparticles. This finding aligns with previous studies on Ag-doped nanoparticles synthesized using rosemary leaf extracts [27]. Compared to chemically synthesized methods, microbial-mediated synthesis typically results in a slight decrease in zeta potential values [28]. This phenomenon is attributed to microbial processes that involve enzyme production, acid formation, and secondary metabolite release, which can alter the pH of the medium and affect zeta potential values. Furthermore, the incorporation of silver into the zinc lattice structure contributes to a reduction in the zeta potential of the nanocomposites due to increased repulsion between the nanoparticles, as observed in previous research [29]. In XRD, the peak shift occurs due to the substitution or interstitial placement of silver within the zinc lattice [30]. The positions of silver within the lattice—whether substitutional or interstitial—determine the direction of the peak shift towards higher or lower angles [26].

A shift towards lower-band position frequencies reflects bond length alterations due to doping sites [31]. The absence of chemical bending between Ag-ZnO and Ag denotes that silver was successfully doped into the lattice of ZnO [32]. This study underscores the modifications in bonds resulting from Ag doping on the ZnO lattice sites in FTIR analysis. Microscopic analysis also shows that Ag-ZnO nanoparticles are agglomerated and spherical [33,34]. The EDX spectrum of undoped ZnO nanoparticles exhibits Zn peak lines at 1.8 keV and 9.6 keV [35]. A distinct peak at 3 keV in the EDX spectrum confirms the presence of Ag ions [36].

In contemporary settings, many bacteria develop intrinsic resistance mechanisms such as enzyme overproduction, active efflux pumps, and alterations in cell permeability [37]. Determining the minimum inhibitory concentration (MIC) and determining the sub-minimum inhibitory concentration (sub-MIC) are crucial steps that need to be taken to identify effective NP concentrations before assessing quorum sensing inhibition activity. MIC allows bacterial growth without causing microbial death while inhibiting the virulence factors of *P. aeruginosa* by modulating quorum-sensing circuits [38]. *C*. *violaceum* serves as a biosensor for detecting quorum-sensing inhibitors, as it naturally produces a purple pigment known as violacein. The inhibition of violacein pigment serves as an indicator of quorum sensing signal attenuation [39]. AHL (acyl-homoserine lactone) molecules govern violacein production within the quorum sensing system. The ability of nanoparticles to inhibit AHL molecule production correlates with their concentration, highlighting their anti-quorum sensing properties [40].

Pyocyanin, a blue-green pigment and a virulence factor regulated by quorum sensing, induces oxidative stress in hosts, contributing to cellular damage in cystic fibrosis patients [41]. The inhibition of pyocyanin was evident from the change in its blue-green color, with inhibition levels correlating with NP concentration [42]. The crystal violet assay is a reliable method that can be used for assessing the inhibition of biofilm formation, a critical factor contributing to the resistance mechanisms of *P. aeruginosa* [43]. NPs effectively reduce biofilm formation without affecting planktonic cells compared to the control, demonstrating the dose-dependent inhibition of biofilm formation [44]. EPSs including nucleic acids, extracellular DNA, proteins, and polysaccharides are crucial for biofilm formation, serving as a protective shield for bacteria in the environment [45].

Quorum sensing (QS) signals regulate EPS production and biofilm formation in bacteria, with EPSs providing mechanical and structural stability to biofilms. The inhibition of QS signaling using NPs reduces EPS synthesis, weakening biofilm integrity. Biogenically synthesized NPs can penetrate bacterial cells, interact with QS molecules, and disrupt EPS production. Unlike antibiotics hindered by biofilm barriers, nanosized metals can penetrate biofilm matrices and inhibit EPS production [46]. Swarming motility is crucial for *P. aeruginosa* as it regulates bacterial attachment to surfaces, facilitating biofilm formation and infection [47]. Increasing concentrations of ZnO nanoparticles result in reduced swarming motility, indicating the disruption of the motility mechanism due to biogenic ZnO synthesis [48]. Higher nanoparticle concentrations significantly diminish swarming motility compared to lower concentrations, with the degree of reduction being dependent on the nanoparticle dosage [18]. Proteases play a critical role in exotoxin secretion and are regulated by QS systems. Different concentrations of NPs result in varied clear zones around the well on agar plates, indicating the inhibition of protease production [49]. The reduction in zone formation on skim milk agar is dependent on the concentration of NPs used [43]. Therefore, biogenically synthesized Ag-ZnO NCs demonstrate excellent inhibition, effectively reducing the virulence factor of the MDR pathogen without compromising bacterial growth. Thus, based on current and previous research, it is confirmed that the synthesis of nanoparticles using bacterial cells will be one of the most effective strategies for the targeted control of bacterial pathogens, owing to its interaction with the pathogen cell membrane component and transport systems [42,50].

## 4. Materials and Methods

### 4.1. Reagents

Zinc acetate dihydrate (Zn (CH_3_COO)_2_(H_2_O)_2_) and silver nitrate (AgNO_3_) were purchased from Sigma Aldrich, India. Sodium hydroxide, crystal violet, and Congo red were purchased from High Media, Bangalore, India. The procured chemicals were of analytical grade.

### 4.2. Microorganisms

Multidrug-resistant (MDR) *Pseudomonas aeruginosa* was properly obtained from K.A.P. Viswanathan Government Medical College, Tiruchirappalli, India. *Chromobacterium violaceum* MTCC2656 was acquired from the Microbial Type Culture Collection (MTCC) in Chandigarh, India. The test and biomarker strains were cultured in tryptic soy broth (TSB) and Luria–Bertani (LB) broth and incubated at 37 °C. Later, the collected strains were preserved in a cool condition for further study.

### 4.3. Isolation, Identification, and Screening of Metal-Tolerant Bacteria

A soil sample was obtained from a metal-polluted site near BHEL (Bharath Heavy Electricals Limited), Tamil Nadu, India (10.8282° N 78.6932° E). Metal-tolerant bacteria were isolated using an enrichment technique with slight modifications based on the method outlined by [51]. Then, 10 g of the soil sample was introduced into a sterile conical flask containing 90 mL of minimal media broth supplemented with zinc chloride (5 mM) and silver nitrate (5 mM) and incubated at 37 °C for 72 h while being shaken at 100 rpm. From the culture, 0.1 mL of fresh inoculum was aseptically transferred to nutrient agar and allowed to incubate at 37 °C for 24 h. Following incubation isolated bacterial strains (2.3 × 10^6^ cells) were added to 100 mL of deionized water spiked with metal substrates and screened for nanocomposite synthesis based on turbidity formation. The isolated bacteria demonstrating potent nanocomposite synthesis capabilities were designated as SASAK.

Subsequently, the isolated strain underwent partial identification using the 16S rRNA sequencing method, as described by [52], employing forward (5′-AGAGTTTGATCMTGGCTCAG-3′) and reverse (5′-AGAGTTTGATCMTGGCTCAG-3′) primers. Sequences were analyzed using BLAST and deposited in the NCBI GenBank under accession number MH885570. The 16S rRNA sequence of the isolated strain was extracted from nucleotide databases and aligned using MUSCLE software. A phylogenetic tree was constructed using CLC MAIN Workbench 4 software, employing the neighbor-joining distance method to assess the similarity between the isolated strains and others in the database.

### 4.4. Bacterial-Mediated Synthesis of NCs

The bacterial-mediated synthesis of pure nanoparticles and Ag-ZnO NCs was performed using the protocol outlined in [53]. The isolated bacteria were cultivated in 50 mL of nutrient medium and incubated at 37 °C for 24 h. Subsequently, 50 mL of the overnight culture was diluted with 150 mL of sterile deionized water and further incubated for 24 h. The bacterial solution was serially diluted and allowed to achieve synchronous growth by adjusting the pH to 6.5 using 0.4 M NaOH. Next, 0.1 M zinc acetate dihydrate and varying concentrations of silver nitrate (1%, 2%, and 3%) were prepared and added to the bacterial culture solution. The culture was then placed on a rotary shaker at 37 °C for 24 h. The appearance of a brown precipitate in the medium indicated the biotransformation process. After incubation, the transformed solution was centrifuged at 5000 rpm for 20 min, and the precipitate was collected and dried in a Petri dish in a hot air oven for 3 h. The obtained NCs were further calcined at 200 °C in a muffle furnace for 3 h. A similar procedure was followed for synthesizing ZnO nanoparticles without adding silver nitrate. The nanocomposites were then ground into powder using an agate mortar and pestle and stored at room temperature for further analysis.

### 4.5. Characterization of ZnO NPs and Silver-Doped Zinc Oxide NCs

The optical properties of the nanocomposites were verified using UV-VIS-NIR spectroscopy (Cary 5000(1.12), New Delhi, India), measuring absorbance across the range of 300–700 nm. Differences in functional groups of the nanocomposites were identified using FT-IR spectrophotometry (SYMADZA-8400, New Delhi, India). The crystalline structure of the NCs was analyzed using XRD, utilizing an instrument operating at a 30 mA current and a 40 kV voltage (BRUKER-binary (V3), New Delhi, India). Zeta potential analysis was employed to assess the potential stability and surface charge of the nanocomposites within the range of ±30 mV (Malvern). The average size of the NCs was determined using a particle size analyzer (Particulate system). The structural characterization of NCs was conducted using FE-SEM and HRTEM. EDX analysis provided elemental identification and quantitative compositional information on the nanocomposites.

### 4.6. Antibiotic Susceptibility Test Against the Pathogenic Strain of P. aeruginosa

The drug susceptibility testing of multidrug-resistant *Pseudomonas aeruginosa* was conducted following the previously outlined method [54]. A 0.1 mL aliquot of an overnight culture of the test pathogen (10^−4^ cells/mL) was spread onto sterile agar plates. Subsequently, seven different standard antibiotic discs, gentamicin, streptomycin, kanamycin, ciprofloxacin, piperacillin, penicillin G, and ertapenem, were aseptically placed on the agar plates, which were then incubated for 24 h at 37 °C. The results were interpreted based on the presence and size of zones of inhibition surrounding each antibiotic disc.

### 4.7. Minimum Inhibitory Concentration (MIC) and Sub-MIC Determination of Ag-ZnO NCs

The minimum inhibitory concentration (MIC) and sub-MIC levels of NCs were determined using the broth tube dilution technique outlined earlier [45]. An aliquot of sterile test tubes was taken, and each tube containing 10 mL of sterile TSB medium supplemented with various concentrations of Ag-ZnO NCs (12.5, 25, 50, 100, 200, 400, 800, and 1600 µg/mL) was inoculated with 0.1 mL of test pathogenic strain (approximately containing 3.8 × 10^5^ cells/mL). Positive controls (without nanocomposites) and negative controls (with colistin at 0.5 µg/mL) were also included, and all tubes were incubated at room temperature with shaking. After incubation, a UV spectrophotometer assessed bacterial growth inhibition by measuring optical density at 600 nm. The MIC was defined as the lowest concentration of NCs that completely inhibited the growth of multidrug-resistant *Pseudomonas aeruginosa*. The maximum doses at which the nanocomposites showed no inhibitory action against the test pathogen were referred to as sub-MIC.

### 4.8. Anti-Quorum Sensing Activity of NCs

#### 4.8.1. Violacein Inhibition Assay

A qualitative analysis of violacein inhibition was performed according to the procedure outlined in [55]. Briefly, the overnight culture of *C*. *violaceum* (2.4 × 10^6^) cells was added to 1 mL of LB broth and spiked with Ag-doped ZnO NCs (12.5, 25, 50, and 100). Tubes were incubated for 48 h at a specific temperature. Violacein pigment was quantified by measuring the absorbance value at 600 nm.

#### 4.8.2. Swarming Assay

The swarming motility of *P. aeruginosa* was assessed using the methodology outlined in [47]. Sterile swarming agar plates were prepared by mixing nutrient broth (0.8%), glucose (0.5%), agar (0.5%), and the sub-MIC concentration of NCs, as described. Plates without nanocomposites served as negative controls. Subsequently, fresh cultures of *P. aeruginosa* were inoculated at the center of each agar plate and incubated at room temperature. The swarming behavior of bacteria on plates containing ZnO NPs and Ag-doped ZnO NCs was observed, compared, and documented.

#### 4.8.3. Pyocyanin Assay

The pyocyanin assay was conducted with minor adjustments, as described in [48]. *P. aeruginosa* was inoculated into nutrient broth supplemented with pure ZnO NPs (50 µg/mL) and Ag-ZnO NCs (12.5, 25, 50, and 100 µg/mL) and then incubated under specific conditions (24 h at 37 °C). A control tube containing the pathogen without nanocomposites was also prepared. After incubation, pyocyanin was extracted by centrifuging the samples for 20 min at 5000 rpm. Next, 5 mL of supernatant was mixed with 3 mL of chloroform, and the lower chloroform layer was re-extracted with 1 mL of 0.2 M HCl. The resulting pink-colored solution was obtained, and the pyocyanin content was quantified spectrophotometrically at 520 nm. The final amount of pyocyanin was calculated by multiplying the OD values obtained at 520 nm by 17.072. The percentage of pyocyanin inhibition was determined using the following equation (Equation (4)), according to [56].

(4) $$Pyocyanin\;inhibition\left(\%\right)=\frac{Control\;{OD}_{520}-Test\;{OD}_{520}}{Control\;{OD}_{520}}\times 100$$

#### 4.8.4. Congo Red Agar (CRA) Assay

To assess the impact of nanocomposites on exopolysaccharide (biofilm) production, the CRA (Congo red agar) test was conducted following the procedure outlined in [57]. CRA plates were prepared using brain heart infusion (BHI) broth supplemented with agar (10 g/L), sucrose (50 g/L), and Congo red dye (0.08 g/L), with ZnO (50 µg/mL) and varying concentrations of Ag-ZnO nanocomposites (12.5, 25, 50, and 100 µg/mL). Plates without nanocomposites served as controls. Then, *P. aeruginosa* strains were streaked onto the CRA plates and incubated until black-colored colonies appeared, indicating biofilm formation on the agar surface.

#### 4.8.5. Crystal Violet Assay

The antibiofilm efficiency of nanocomposites was examined according to the procedure of [43] with some modifications. Afterwards, 10 mL of nutrient broth was taken in sterile test tubes and seeded with 10^6^ cells of *P. aeruginosa*, along with the absence or presence of NCs with different concentrations. After incubation, free-floating cells were removed and washed with a phosphate saline buffer. Then, the remaining part was air-dried, and the attached part of the biofilms was stained with 1% crystal violet. Later, the tube was washed with distilled water to eliminate the unwanted stain. The biofilm attached to the tube was eluted with ethanol, and the OD value was noted at 570 nm. The inhibition of the biofilm was determined using the following equation (Equation (5)).

(5) $$Percentage\;of\;inhibition=\frac{Control\;{OD}_{570}-Test\;{OD}_{570}}{Control\;{OD}_{570}}\times 100$$

#### 4.8.6. Effect of NCS on Exopolysaccharide (EPS) Production

The EPS assay was carried out to assess the level of EPS production in MDR *P. aeruginosa* treated with and without NCs [58]. Aliquots of sterile test tubes containing LB broth spiked with *P. aeruginosa* culture with the presence and absence of NCs were taken. After incubation, the biofilm was developed in tubes, and planktonic cells were removed through centrifugation (10 min for 10,000 rpm). Then, 0.5% NaCl was added to the solution, vortexed, and transferred to sterile test tubes. Finally, an equal volume of phenol was added to that solution tube with 5 volumes of sulfuric acid and incubated in the dark for 1 h. The EPS production inhibition percentage was calculated by measuring the OD using a UV spectrophotometer at 490 nm (Equation (6)).

(6) $$EPS\;Production\;inhibition\left(\%\right)=\frac{Control\;{OD}_{490}-Test\;{OD}_{490}}{Control\;{OD}_{490}}\times 100$$

#### 4.8.7. Protease Assay

The effect of Ag-ZnO NCs on extracellular protease production using *P. aeruginosa* was analyzed according to the procedure described earlier by [59]. The test pathogenic strain was allowed to grow on LB broth with nanocomposites ranging from 12.5 to 100 (µg/mL) and incubated. Later, the supernatants were collected through centrifugation and a filtration process. Before being incubated, the supernatants (10 µL) were added to the well on the center of the skim milk agar plates containing the following compositions (g/L): casein enzymic hydrolysate (5), yeast extract (2.5), skim milk powder (1), glucose (1), and agar (10). The inhibition of bacterial protease activity using NCs was measured by calculating the diameter (cm) of the clear zone around the well.

### 4.9. Statistical Analysis

All experiments were conducted in triplicates to validate reproducibility. The data are presented as mean ± standard deviation (SD).

## 5. Conclusions

The biological synthesis of nanocomposites has emerged as a cost-effective, safe, and environmentally friendly alternative to conventional physical and chemical methods. Utilizing bacteria for nanocomposite synthesis offers advantages in terms of ease of handling and genetic manipulation, alongside demonstrating excellent anti-quorum sensing activity against MDR *P. aeruginosa*. The sub-MIC concentration of 2% Ag-ZnO NCs effectively attenuates virulent factors such as pyocyanin, swarming motility, protease activity, biofilm formation, and violacein pigment production in *C. violaceum*, while maintaining minimal impact on bacterial growth. Ag-ZnO NCs show promising potential as anti-quorum sensing agents against pathogenic MDR *P. aeruginosa*. These metal-doped nanocomposites likely interact with quorum-sensing molecules or receptors, thereby inhibiting the expression of virulence genes in *P. aeruginosa.* Furthermore, the combination of silver and zinc oxide nanoparticles synergistically enhances their effectiveness in inhibiting the virulence factors of *P. aeruginosa* compared to undoped ZnO NPs alone. This synergistic effect underscores the potential of nanocomposites in combating MDR pathogens, warranting the further exploration of their mechanisms in future research endeavors.

## Data Availability

The data presented in this study are available from the corresponding author upon reasonable request.
